# Agricultural Robotics for Field Operations

**DOI:** 10.3390/s20092672

**Published:** 2020-05-07

**Authors:** Spyros Fountas, Nikos Mylonas, Ioannis Malounas, Efthymios Rodias, Christoph Hellmann Santos, Erik Pekkeriet

**Affiliations:** 1 Agricultural University of Athens, Iera Odos 75, 11855 Athens, Greece; nmylonas@aua.gr (N.M.); gmalounas@aua.gr (I.M.); efthimisr@yahoo.gr (E.R.); 2 Fraunhofer IPA, Nobelstr 12, 70569 Stuttgart, Germany; christoph.hellmann.santos@ipa.fraunhofer.de; 3 Wageningen Plant Research, Wageningen University and Research, P.O. Box 644, 6700 AP Wageningen, The Netherlands; erik.pekkeriet@wur.nl

**Keywords:** crops, autonomous vehicles, field operations, perception, execution

## Abstract

Modern agriculture is related to a revolution that occurred in a large group of technologies (e.g., informatics, sensors, navigation) within the last decades. In crop production systems, there are field operations that are quite labour-intensive either due to their complexity or because of the fact that they are connected to sensitive plants/edible product interaction, or because of the repetitiveness they require throughout a crop production cycle. These are the key factors for the development of agricultural robots. In this paper, a systematic review of the literature has been conducted on research and commercial agricultural robotics used in crop field operations. This study underlined that the most explored robotic systems were related to harvesting and weeding, while the less studied were the disease detection and seeding robots. The optimization and further development of agricultural robotics are vital, and should be evolved by producing faster processing algorithms, better communication between the robotic platforms and the implements, and advanced sensing systems.

## 1. Introduction

Major technological advancements in agriculture have drastically transformed several processes, both in crop and livestock production systems, during the last decades. These advancements are mainly related to the minimization of operational and production costs, reduction of environmental impact and optimization of the overall production cycle. Focusing on crop production, a series of optimization models and software tools have been developed so far on field-operation level. This progress, in parallel with the technological advancements and equipment in field machinery, has provided radical solutions to several challenges that modern farmers face. In crop production systems, one of the most significant issues is connected to human labour-intensive operations. These are, mainly, field tasks (such as sensitive fruits harvesting and intra-row weed control) that are more difficult to be executed by traditional field machinery, and human workers are employed. This has brought the increased need for autonomous tractors and robotic platforms to be used in the crop field operations, currently developed at research stage [1]. 

Field operations in agriculture are quite complex, and various issues should be addressed to allow an effective transition towards the robotics era. To build a robotic solution, an overall system analysis of the field operation should be conducted, together with a cost–benefit analysis [2,3]. Such a system should comply with very specific requirements, such as lightweight, small size, autonomy, intelligence, communication, safety, and adaptability, to execute the potential task effectively [4]. The relatively smaller size of autonomous machines, compared to conventional tractors and implements, contributes to the reduction of the soil-related issues and in particular soil erosion and soil compaction, that was provoked by large and heavy modern farm machinery [5]. To automate these field operations, a decomposition of these tasks should be performed to transform them into discrete robotic functionalities. This can be achieved by classifying the agricultural tasks into deterministic (tasks that can be designed and optimized, in advance) and reactive (tasks that are associated with behaviors that should deal with unexpected conditions) [6]. 

The main challenges that agricultural robots face are associated with both universal and task-specific issues. Universal issues are related to terrain assessment [7,8], route planning [9,10], safety issues, especially focusing on human detection [11], and fleet of robots [11,12,13]. Task-specific issues are related to specifications related to crop architecture, crop or pests detection and classification, and precise application of inputs. Most of these issues are related to the vision system, the robotic actuation system, the navigation system in semi-structured agricultural environments, and the intelligence to control both the robotic platform and the implement. 

There have been several reviews focusing on agricultural robots for field operations based on specific tasks (such as agricultural navigation, transplanting/seeding, pruning, weed control, harvesting, traceability and multi-robot interactions) and systems assessment [14]. One study reviewed a group of field operations applied only in open arable farming systems [15], while the digital perspective of mainly commercial agricultural robots has been reviewed as well [16]. Finally, a short review based on the methodological approach, the field operation and the farming environment has been presented, under the demonstration of an emulation tool for agri-robotics [17]. As weed control robots are among the most developed agricultural robots so far, a review has been conducted regarding only autonomous weed control systems [18], while there has also been a crop-specific literature review about robotic systems applied to strawberries [19]. Even though the scientific contribution of these reviews is highly important, the contribution of this study is to provide a holistic review of agricultural robotic systems that execute all the main field operations, for all cropping systems and in both open-field and greenhouse environments.

The aim of this paper was to study the specific issues related to main field operations in crop production. More specifically, the main challenges leading to the concept of the current review are: (i) to identify and emphasize the currently existing robotic systems developed for each field operation, regarding different crop production systems; (ii) to provoke further development and optimization of existing agricultural robotic systems in general and, more specifically, the development of smarter perception and actuation systems; and (iii) to propose the key challenges and directions for future agricultural robotic developments. 

This review paper covers both scientific and commercial agricultural robotic systems applied in various field operations (such as weeding, seeding, disease detection, crop scouting, spraying and harvesting) for different farming environments (arable, greenhouses, vineyards and orchards). The structure of the present work is as follows: the followed framework in this review is showcased in Section 2. In Section 3, the operational classification and analysis are both presented and allocated in the main field operations sub-sections. Finally, the overall discussion of the review is shown in Section 4, followed by Section 5 where general conclusions are underlined. 

## 2. Review Framework

**Figure 1:**
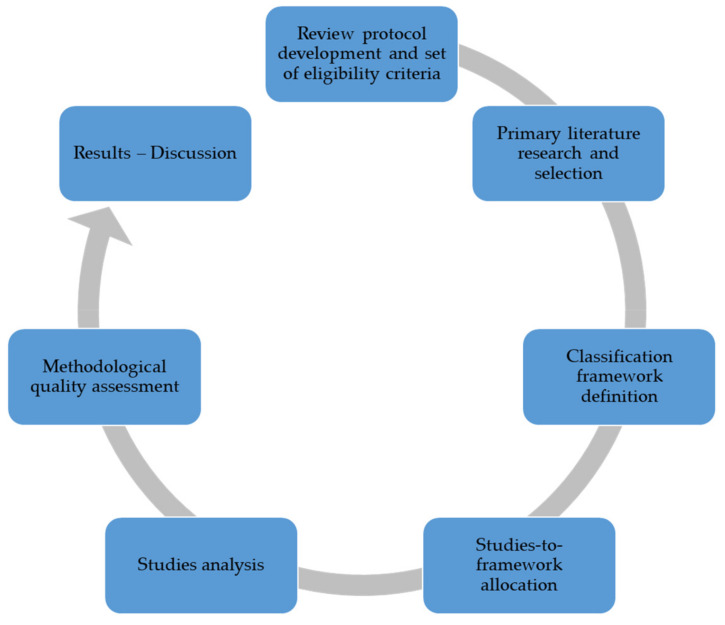
Steps of review methodology.

In the pre-processing stage of this review, a series of theoretical considerations were taken into account. Throughout the analysis, a series of steps were established, as presented in Figure 1: (i) development of the review protocol regarding the eligibility criteria; (ii) search for research studies and commercial resources, and selection of the ones satisfying the eligibility criteria; (iii) definition of the classification framework to be applied in the literature review, in order to classify the material and build the structure; (iv) selection of studies to be included in each category of the classification framework; (v) analysis of the selected studies; and (vi) representation of results by comparing studies.

Regarding the eligibility criteria, the studies that are related to robotic systems applied in crop production systems have been included as a wide boundary of this study. More specifically, the included references should be related to robotic systems that conduct specific field operation(s) in crop production systems in agriculture. The referenced robotic systems may have been developed in various environments (i.e., open-field, greenhouses, laboratory, etc.). Hence, they should be applicable to any in-field operation, excluding out-of-field transportation operations.

In addition, a number of literature-related eligibility criteria are set to be followed. Those are: (1) the work should be published in the English language; (2) the included studies should be research articles published in scientific journals, conference proceedings, or other sources (commercial websites, research projects, etc.); (3) they should have been published within the last 20 years (without including the current year), as any technology used prior to this is probably obsolete today. Publications in journals that are not research articles (such as reviews) are out of the scope of this review and they are excluded. 

The included studies have been withdrawn from open online sources (such as open-access journals, websites, conference proceedings, etc.) and by a systematic literature review in various electronic depositories, namely Web of Science, ScienceDirect, Scopus and SpringerLink. The primary literature research was conducted at the end of 2019. Thus, any newly emerging publication will have been possibly missed. Due to the nature of the reviewed field, grey literature (technical/research reports, doctoral dissertations, etc.) have been knowingly permitted by the authors in order to cover as much as possible in this review.

## 3. Operational Classification and Analysis

The existing literature of robotic systems in agricultural field operations was classified according to major field operations, as follows: (1) weeding, (2) seeding, (3) disease and insect detection, (4) crop scouting (plant monitoring and phenotyping), (5) spraying, (6) harvesting, (7) plant management robots, and (8) multi-purpose robotic systems [4]. Given that the current literature is rather wide, a brief summary of the most important literature, based on scientific and commercial resources, has been allocated to these categories and presented below.

### 3.1. Weeding Robotic Systems

Weeding is one of the most repetitive, tedious and time-consuming activities within the crop production cycle. Especially for developing countries and smallholder farms, weed management accounts for more than 40% of the labor effort [20]. Besides being one of the most difficult tasks, it is one of the most costly, including among other factors significant labor cost, especially in crops that are highly labor-demanding. Moreover, it is a field operation that has a substantial negative impact on the environment, as large quantities of herbicides are used to keep the field weed-free, to promote plant health and yield. As an example, global estimated loss potential in rice, wheat and maize indicates that weeds account for 46.2–61.5% of potential yield losses, and 27.3–33.7% of actual yield losses caused by all pests together [21]. For all the above-mentioned reasons, significant attention has been given to weeding robots both by private companies and academia. A number of such weed robots have been presented in a short review [22], while in the current study a wider review has been conducted regarding the major field operations, including weeding robots.

Traditionally, there have been two approaches for weeding; namely, mechanical weeding, and chemical weeding. Mechanical weeding describes the task of destroying weed plants, mainly by plucking them out, burning them or cutting them. For each of the three above-mentioned methods of mechanical weeding, a variety of tools are available. In order to remove the weeds by mechanical means, the system should first detect the row that it will work on, and then cut or pluck out the weeds. The precision in row detection recorded with mechanical weeding robots was less than 25 mm [23,24], followed by other systems with precision less than 6 mm, [25,26] or higher, up to 3 cm [27,28]. In addition, the performance of the weed removal (expressed also as weeding efficiency) has been evaluated in systems that presented rates higher than 90% [24,29], and 65% and 82%—for two different presented methods [30]. Apart from those, there are a series of mainly commercial mechanical weeding robots that have no performance metrics provided [31,32,33,34].

On the other hand, chemical weeding refers to spraying the weeds with herbicides that have a toxic effect, thus eliminating the weeds, and it has been popular because it requires considerably less labor. Herbicides manufacturers have also developed active ingredients that are only toxic to weeds and not crops, allowing for extensive spraying over the entire field. However, a number of chemical substances, especially in the EU, have already been restricted in their use, or are going to be restricted in the near future, due to the negative impact on the environment and human health. In addition, weeds have already developed chemical resistances, making them harder to eradicate. The main solution proposed to reduce the pollution caused by herbicides and delay the appearance of resistant weeds is selective/spot spraying, spraying only the weed and not the entire field. This would require even more working hours to be spent on weeding. On the bright side, recent developments in computer vision technologies have allowed manufacturers to automate that process, and integrate high-level software into spraying/weeding robots.

Similar to mechanical weeding robots, the effectiveness of the system for spotting weeds is among the most critical performance metrics for the chemical weeding robots. By using a Drop on Demand (DoD) system—a system that detects the weeds within the plant row and selectively shoots droplets of herbicide onto those weed leaves—100% effectiveness was achieved, which is the highest among chemical robots [22,35]. In parallel, 98% and 89% detection accuracy were presented in a double experiment of a different vision-based weeding robot [36]. Finally, effectiveness (weed detection and destruction) of more than 85% under ideal conditions is underlined by the EcoRobotix weeding robot [37]. While a series of commercial chemical weeding robotic systems are available, almost no performance metrics exist [38,39,40,41]. The perception sensors used for weeding offer similar levels of performance. All robots make use of a camera, where RGB (Red–Green–Blue), infrared (IR) and web cameras are the most commonly used. There also exist sensors that allow the robot to identify its surroundings and its relative position to the weeds, using acoustic distance sensors, gyroscopes, laser range finders, Inertial Measurement Unit (IMU), etc. Table 1 outlines the most significant chemical and mechanical weeding robotic systems with their main characteristics in terms of perception sensors, level of weed detection, control method and performance rates (where they exist).

To conclude, weeding robots have already been under development over the last years, and there are also a number of commercial robots on the market. Most of the major problems related to the existing weeding robots are being dealt with—mainly, the use of recent advancements in computer science (e.g., deep learning) and the integration of more accurate navigation systems (e.g., RTK GPS). What remains to be done is to improve their performance, increase working area and working speed, and increase the accurate detection of weeds for selective spot spraying. There are a few key issues that should be taken into account; i.e., how the existing robotic systems could perform on crops with similar operational requirements, to expand their potential use and the overall development of weeding robots in crops with low competitiveness towards weeds, such as specific vegetables. In most weeding robots, the vision sensors are currently used in combination with distance identification sensors. This, though, could be potentially enhanced by even higher resolution weed detection sensors for selective spot spraying. Generally, existing robots focus on above-ground weed detection. Possible solutions for future weeding robots may include the detection of weeds in their primary growth, when the weed sprouts are still below the ground. Such systems could be equipped with sensors measuring, for instance, the soil electric conductivity.

### 3.2. Seeding

Seeding is one of the fundamental tasks in a crop production cycle and contributes significantly to the labor cost. Seeding machinery has evolved to offer precision seeding solutions. Since recent publications have shown the importance of plant densities to a high yield [43], precision seeding has gained more interest in the scientific community, which as a result has led to the development of several seeding robots in recent years (Table 2).

The main performance metrics presented in the existing literature are connected either to accuracy rates (%), regarding the number of seeds that have been accurately placed, or to position errors. The main targeted crops for the existing reported seeding robots are mainly cereals, with high accuracy rates reported regarding wheat and rice crops [44,45]. On the other hand, an autonomous seeding robot more suitable for wet-fields has been proposed, but with no performance metrics [46]. Finally, there has been progress in commercial seeding robots made by Fendt [47,48], which can be used as an intelligent fleet of seeding robots or individual units. The most important information for seeding is the depth and the position that the seed is going to be planted. As a result, force, pressure, angle and displacement sensors are used in combination with wheel encoders, compasses and displacement sensors.

In conclusion, the last years’ seeding robots have been developed for seeding both at com0mercial and research level, but to a limited number due to the complexity of the operation. Even though the current advancements in sensors play a crucial role in facing the issues related to these robots, they should be optimized further by minimizing the errors and maximizing the accuracy rates. There are a few key challenges identified for seeding robots, such as the working speed of the robot, as it is one of the main factors for precision seeding, making its calibration crucial for each robot. Moreover, the need to improve speed and accuracy, with better vision systems. Better image detection systems that use more advanced algorithms are already under development [50]. Before seeding, such vision systems should be able to identify the soil surface quality and structure, the existence of any weeds, and possible obstacles that may occur. After seeding, the seeding performance should be evaluated, especially in fields with an uneven surface. 

### 3.3. Disease and Insect Detection

Robotic application in disease and insect management (detection and control) has very recently gained attention, due to its complexity and the lack of accurate and efficient conventional systems. Disease detection is an important part of the production cycle, as diseases can cause significant economic damage if not detected during the early stage. Insect detection has not gained a lot of attention, as many insects are located underneath the leaf, inside the buds or even underground, which makes it extremely difficult to accurately detect them and, as a result, monitor them. In Table 3, the main perception features and the highest detection accuracy for each cited work are showcased for a number of crops and diseases.

Disease detection is mainly a visual task, and therefore all robots incorporate a vision-based system. Detection accuracy is defined as the ratio of the identified diseased plants to the total number of the diseased plants. All the reviewed robots use a color camera, which is low-cost and easy to use, with the second most used camera type being a multispectral camera, which is moderately expensive and requires complex higher computational power. In principle, all system configurations with multi-/hyper-spectral sensors were combined with color cameras. At this point, it is worth mentioning that using the more complex system does not always yield the best results. For the detection of powdery mildew and tomato spotted wilt virus (TSWV) in greenhouse pepper plants, using a color camera showed high accuracy, while the use of a multispectral camera achieved an accuracy of 80% and 61% for these two diseases, respectively [51,52]. A different approach was introduced for the detection of *Xylela fastidiosa* in olive trees, fusing a series of sensors [53].

Simpler disease detection robots are based mainly on RGB image inputs. With regards to this, powdery mildew leaf disease has been detected in strawberry plants by a machine vision system, that was based on artificial cloud lighting conditions, and achieved accuracy from 72–95% under two different lighting conditions [54]. Similarly, RGB cameras have been used for *Pyralidae* insect detection on tomato and rice plants with accuracy of about 94% [55]. Moreover, eAGROBOT was used to detect a series of diseases, and presented about 90% accuracy for both cotton plants and groundnut plants [56]. 

To sum up, disease detection robot technology is in its infant stages. From what is available at this point, there are three major problems related to disease detection in robots: (i) the lack of image databases for each disease in order to train the detection/classification models; (ii) the slow image processing, especially when using large volumes of images such as hyperspectral; and (iii) the non-uniform lighting conditions in the field. The lack of available image datasets is gradually being addressed, as open-access agricultural databases are becoming available [57] and new data synthesis methods are presented [58]. At the same time, novel lighting systems are also being proposed for the detection of various diseases such as powdery mildew [54]. As for insect detection, it has not gained attention due to the constraints related to insect features (such as size, color, shape, etc.). Potential future vision systems could detect insects by using multiple ultra high definition cameras, that could identify insects covered among the leaves, underneath or elsewhere on the plant. Even in this case, such an insect detection robot should include high-performance algorithms, given the high volume and size of images, in order to perform accurately. 

### 3.4. Crop Scouting

#### 3.4.1. Plant Vigor Monitoring

Plant monitoring is the oldest practice growers have been performing to ensure high yield and quality. Monitoring plant stress so far has been done by searching for visible symptoms, such as change of color, withering, spots, or any abnormality on the plant leaves or the plant in general. However, as remote sensing technology is advancing, new sensors are becoming available for the grower to monitor plant health and stress, even when it is not visible to the human eye. Nowadays, thermal cameras and infrared thermometers are used to measure canopy temperature [59]. Further, various vegetation indexes, such as the Ratio Vegetation Index (RVI), the Normalized Difference Vegetation Index (NDVI) [60], etc., which provide useful information about plant health, photosynthetic ability and more, are used by combining wavelengths. Moreover, expensive hyperspectral sensors are starting to find applications in plant monitoring systems [61].

Various plant monitoring robots have been developed so far, even though they are in their early maturity level, due to the high cost of the sensors that are utilized. As a result, the main focus has been on high-value crops and how robotic systems can effectively replace humans in monitoring tasks. In Table 4, various plant monitoring robots are presented.

The majority of crop monitoring robots have been developed specifically for orchard/tree crops or vineyards. A crop monitoring robot has been experimentally evaluated in laboratory conditions [62] and semi-structured environments [63], and proved to be highly reliable for the estimation of orchard canopy volume and vigor, by using a series of sensors. Crop monitoring robots for vineyards have been widely developed, even though there are no clear quantified performance metrics. Most of them are commercial crop monitoring robots (such as [64]), or robots that have been developed under EU-funded projects ([65,66,67]). In all cases, a series of vision-related sensors are included, such as various types of cameras (RGB, hyperspectral, multispectral, etc.), and LiDAR sensors are incorporated on the robots, in order to monitor volume, vegetation, and health of the plants. Given that vineyards are usually cultivated in hilly or mountainous terrains, a vineyard monitoring robot deals with a series of challenges related to terrain traversing, mapping, and localization [68]. 

Similar perception systems have been implemented in crop monitoring robots for crops such as Canola, which presented maximum measurement error up to 2.5% [59]. In this specific case, the platform is mounted on a swather. In addition, Earthsense has commercially developed the Terrasentia crop monitoring robot, suitable for various crops such as corn, soybean, wheat, etc.[69].

More generic crop monitoring robots handle agricultural operations, such as soil sampling [70]. CO_2_ concentration levels may have a significant effect on crop growth. Under this scope, a hexapod robot has been developed for the collection of agronomical information, such as soil nutrients and plant growth. The monitored CO_2_ concentration levels were up to 2500 ppm [71]. 

To conclude, plant vigor monitoring robots face challenges such as illumination, background separation, and data manipulation. Various lighting conditions due to weather conditions have been faced so far, by artificial means and by following strict timetables on image collection processing. Background separation is due to neighbor plants that create occlusions in the visual sensors. This problem can be faced either by better algorithms, that could capture these visual interpolations, or by using multiple cameras. The huge amount of data collected is an issue that could be manipulated by faster processing algorithms and more performant hardware. In the future, plant vigor monitoring robots could include smarter vision systems, i.e., high definition cameras that will automatically correct low-light images and apply artificial light where needed, in order to produce uniform image data. 

#### 3.4.2. Phenotyping 

Parallel to the development of plant monitoring robots, phenotyping robots have been under development. Plant monitoring and plant phenotyping have a lot in common, as in both cases, vision systems are used to gather as much information as possible about the plant/fruit under inspection. The difference is that phenotyping robots focus more on morphology, plant growth and traits that contribute to crop performance, while plant monitoring robots focus on the state, health and chemical composition of the plant. Numerous sensors used in phenotyping robots have been reviewed in [72]. The developed phenotyping prototypes are presented in Table 5, for various levels of autonomy. 

Fully autonomous phenotyping robots have been developed in sorghum plant field trials, presenting stalk detection up to 96% [73]. These robots also measure light penetration, leaf erectness, leaf necrosis and GRVI (Green-Red Vegetation Index). Similar autonomous phenotyping robots in sugar beet crop (Mobile and Bettybot) analyze in detail the geometric and colorimetric evolution of the plants [74]. Bonirob, an autonomous phenotyping robot, uses multisensor data fusion to measure population density, plant distribution, plant height, stem thickness, etc. [75].

In addition to the autonomous robots, a fixed-site, fully automated phenotyping platform has been applied in rice, maize and wheat plants [76]. This platform may provide highly accurate measurements on canopy growth throughout crop lifecycles, including plant height root mean square (RMS) error up to about 1.9 cm. In sorghum crop, a tractor-mounted autonomous robot resulted in highly repeatable measurements (such as plant height, width, convex hull volume and plant surface area) that are quite close to the in-field manual measurements [77]. 

Not all of the phenotyping robots are fully automated. Two phenotyping robots are able to work collaboratively in cotton crop, as an autonomous ground vehicle for individual plant data collection, and as a mobile observation tower that inspects the entire field [78], including RMS (Root mean square) errors for plant height less than 0.5 cm, RGB to IR image calibrations of 2.5 pixels, and less than 1 °C for temperature. Finally, another semi-autonomous phenotyping robot has been tested in energy sorghum crop [79]. The average absolute error achieved by the algorithms used in this robot was 13% for stem width estimation, and 15% for plant height estimation. 

In conclusion, plant vigor monitoring and plant phenotyping are two similar tasks, and therefore face identical challenges. Illumination is a major problem, as lighting conditions are constantly changing. So far, the way researchers have dealt with this problem is by using artificial lighting to ensure uniform illumination, and by collecting their data at the same time of the day [77]. Another challenge is the separation of the background, mainly due to occlusions caused by neighbor plants. Various solutions for the above-mentioned problem have been suggested. Some focus on the crop, for example, cultivating and using wider rows, but the major focus is on the technology used, and mainly on improving the algorithms [80] used, as this does not require changes in cultivation practice. The final challenge is to deal with the amount of data generated. Datasets have to be efficiently processed, the workflow has to be automated, especially when data from different types of sensors are gathered, and pipelines that translate data to the consumer must be built. Computer scientists are already introducing better and faster image processing algorithms, and pipelines that could find use in agricultural applications [81,82].

### 3.5. Spraying

Besides weed management, where herbicide sprayers are used, spraying robots can be used for pests (diseases and insects) and also for liquid fertilizers (foliar). As a result, the farmer is exposed to large quantities of those toxic active ingredients no matter the protective measures that are taken. It is, therefore, crucial to introduce spraying robots to prevent possible health hazards. Due to the rapid development of computer vision and artificial intelligence, robotic sprayers feature novel intelligence systems that enable selective spraying, compared to conventional uniform spraying across the crop. Robots could make use of such technologies, reducing agriculture’s environmental impact as well as consumer exposure to pesticides, and preventing the development of resistance to those substances by the targeted organisms. An overview of the spraying robots can be found in Table 6.

Various approaches have been proposed when designing a spraying robot, mainly due to restrictions and possibilities of the operating environment. In greenhouses, two approaches have been identified: robots that move using the greenhouse piping system [83,84], and robots that navigate between rows without being mounted on those pipes [85,86,87]. Moreover, the ability to spray selectively requires an accurate detection system, and therefore advanced sensors need to be mounted on the robot [88,89]. Finally, the spraying system (nozzles) could be mounted directly on the spraying platform [83,84,85,86,87,90], or it could be mounted on a robotic manipulator with various degrees of freedom (DOF). The manipulator used by [91] had 3 DOF, the one used by [89] 6, while the one from [88] had 9 DOF.

Based on the provided results, the execution time of the spraying operation is presented [86,89], while in most cases the effectiveness of the system is reported. The effectiveness of the systems is demonstrated by providing the successful spraying rates, the error of the system [85,87], or both performance metrics [83,88,90]. Finally, only a few of the systems consider real-time detection of the potential spots to be sprayed [88,89,90], while the majority of them do not include real-time detection [83,85,86,87,91].

It can be concluded from the metrics provided in the reviewed papers that spraying robots perform reasonably well, with non-selective sprayers achieving coverage of about 92% and selective sprayers being able to identify and spray 85% of the diseased areas [83]. The speed, however, of selective sprayers is low, with the total spraying time for every seedling in a hectare cultivated with vegetable crops being higher than 30.5 hours [89]. Although, it has to be mentioned that the time needed can be considerably less since not all plants need to be sprayed. Some of the solutions suggested to decrease the time needed for spraying involve using more spray nozzles or more than one platform, and increasing the speed of the detection algorithms. All these are directly connected to the further development of detection sensors, in order to reach even better accuracy rates in the future.

### 3.6. Harvesting Robotic Systems

Harvesting is one of the most labor-intensive and repetitive tasks, while it is part of all production cycles in agriculture. As a result, several robotic systems have been developed by private companies and universities/research centers to automate this procedure.

There are two types of robotic harvesters: bulk (every fruit/vegetable is harvested) and selective (only the ripe/ready to be harvested fruit are collected). In this review, the focus is mainly on selective robotic harvesters, as this has attracted the most attention from research development. The two major performance metrics of selective robotic harvesting are picking speed and picking rate (i.e., the number of fruits successfully picked out of the number of ready to be harvested fruit [92]). Harvesting must be performed within a certain time slot when the crop is mature, and the majority of the crop has to be harvested and all the above executed without damaging the crop and the plant.

The majority of the harvesting robots focus on strawberries, a high-value crop, that suffers from a high production cost mainly due to labor cost, particularly during harvesting [93,94]. For strawberries, the fastest picking robots were the strawberry harvesting robots in [92,95], with picking speeds of 7.5 and 8.6 seconds per strawberry, respectively, and the Berry 5 robot from Harvest Croo, with a claimed picking speed of 8 seconds per fruit [96]. Other strawberry harvesting robots achieved picking speed up to 10 s/fruit [97] and 11.5 s/fruit [98]. However, having a high picking speed is not enough, as a high picking rate is also important. Having unharvested fruit means that the grower will have to manually harvest these fruits, which is not additional labor compared to manual harvesting, but it is still labor which adds to the total labor cost. The highest picking rate presented was 86% [64]. A range in picking success rates, from 0% up to 64%, has been shown for various categories to evaluate robots’ harvesting performance [99]. It is worth mentioning that for strawberry harvesting, it appears to be a trade-off between picking speed and picking rate, as fast picking speeds are accompanied by poor picking rates and vice versa. Apart from the abovementioned robots, there is, also, a wide variety of commercial strawberry harvesting robots with no performance metrics provided. Dogtooth technologies developed a selective picking robot for table-top growing systems [100], Agrobot developed the E-series, an autonomous selective strawberry harvester with 24 robotic arms [101], while Octinion presented Rubion harvesting robot [102].

Other crops that have gained attention are apples and tomatoes. The apple fruit has attributes that make it easy to harvest robotically. Red varieties are easy to distinguish among the canopy, while the hard nature of the fruit makes it easier for grippers to harvest it without damaging it. The fastest apple harvesting robots have reported picking speeds of 7.5 s per apple [103] and 9 s per apple [104], while the highest picking rates showcased were 89% [105] and about 90% in high-density apple orchards [106]. Tomatoes are cultivated worldwide, and in terms of vegetable crop production, the tomato only falls behind the potato. The fastest tomato harvester had a picking speed of 23 s per tomato [107], while the most reliable one had a picking rate of 87% [108]. In cherry tomatoes, the picking speed was 8 s per tomato bunch without including the moving time [109]. Metomotion [110] and Root-AI [111] have their own selective commercial tomato harvesters, as well.

Similar to apple robotic harvesters, other references regarding various fruits’ robotic harvesters are included in this study. Citrus harvesters, such as the commercial one by Energid, manage to collect each orange in 2–3 s [112]. Another fruit that has gained attention is cherries, which are also a fruit that can be easily detected by robotic harvesters, with picking speeds up to 14 s per fruit having been shown in cherry orchards [113]. Moreover, Agribot, a manually driven fruit robotic harvester, needs two seconds to grasp and detach each fruit [114]. 

Vegetables, in general, represent a promising group of crops in which robotic systems could be implemented. Considering this, a series of different robotic harvesters have been developed. A cucumber robot claimed picking rate of 80%, while its average picking speed was 45 s per cucumber [115]. For eggplants, the picking rate was about 62%, while it needed 64 seconds to harvest an eggplant [116]. The Sweeper robot, that was an EU-funded project, is a selective sweet peppers harvesting robot that, achieved an average picking speed of 24 s/pepper, based on the project’s preliminary results [117]. In asparagus crop, the achieved picking speed was 11.9 s per asparagus, when the robot harvested two asparagus at the same position, while in the case that there was only one asparagus to be harvested in each position, the picking speed was 13.7 s/asparagus [118]. Sparter, an asparagus harvesting robot, is also commercially available by Cerescon [119]. 

Apart from the aforementioned vegetables and fruits, there are certain vegetables that require different harvesting principles due to the challenging features they have, e.g., size and weight. As a result, kinematic analysis and simulation have attracted interest, in order to allow the robotic harvesting of heavy agricultural products, such as pumpkin and cabbage [120]. Regarding fruits, melon and watermelon are representative with similar characteristics. For a melon robotic harvester, the picking rate was about 85% [121], while Stork, that was developed for watermelon harvesting, achieved a picking rate up to 67% [122].

Regardless of the aforementioned categories of robotic harvesters, certain systems refer to miscellaneous crops. Demeter system, designed for the harvesting of alfalfa and sudan (*Sorghum vulgare sudanese*), achieved harvesting speed up to 2 ha·h^−1^ [123]. The performance of a mushroom (*Agaricus bisporus*) robotic harvester regarding its picking success rate was found to be about 70% [124]. In this case, a requirement was to keep scrap rates (i.e., mushrooms that have been damaged in a way that does not meet quality standards) between 5% and 10% (human performance), however, the scrap rate of the robot was about 22%. 

A summary of the main reviewed harvesting robots, as well as the highest picking speed and picking rate per crop, are given in Table 7. A direct comparison between robots designed for different crops is irrelevant, as the requirements for each robot are vastly different. 

From the reviewed harvesting robots, two were tractor-mounted—the Sparter asparagus harvester from Cerescon and the robotic apple harvester [104]—one was manually driven—the strawberry harvester [95] —and the rest were autonomous. Regarding picking mechanisms, there are two main systems—grippers and suction devices. Grippers are devices that consist of mostly rigid joints and links, and are used to pick up and hold an object, or to exert forces on an object causing rotation with respect to the manipulator reference frame [125]. Suction devices use vacuum to singulate fruit/vegetable, and pull, hold or twist it. For detaching the fruits various approaches have been explored, such as removing the fruit/vegetable by cutting the peduncle using the gripper’s fingers [101], cutting the peduncle using blades mounted on the fingers [92,118] and using the gripper or vacuum suction tool to pluck off the fruit [107,126]. There was one solution, though, from Energid industries, focused on citrus fruit, which did not use any grabbing tools. Instead, it made use of extending rods which aimed at the stem, cutting it and causing the citrus to detach [112]. Prior to the fruit harvesting, it first needs to be localized/identified. The main sensors used for the localization of the fruit were RGB cameras, which in some cases were combined with time of flight sensors [117], infrared sensors [95,101] or laser sensors [109]. Cerescon with its Sparter robot had a completely different approach for localizing the fruit, due to the particular cultivation technique of white asparagus (the asparagus grows in sand beds under the soil) [119]. As a result, proximity sensors were used instead of cameras. Finally, the manipulator’s degrees of freedom ranged from 2 to 7 depending on the crop and the adopted solution, with 5 or 6 of freedom being the most common solution.

**Figure 2:**
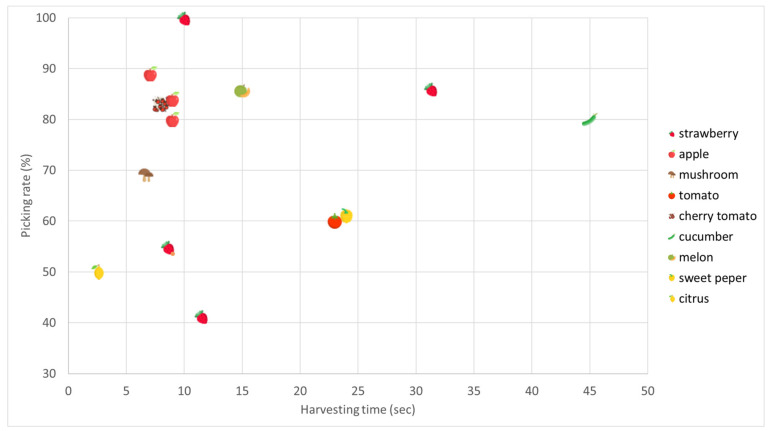
Global illustration of the reviewed robots’ overall performance.

A global illustration of the overall performance of each reviewed robot, where the performance metrics are presented, can be found in Figure 2 (multiple robots for each crop). The figure is not provided for a direct comparison but to get a better understanding of how variation in shape, size and fruit characteristics, such as firmness, affect the picking rate and picking speed.

To conclude, harvesting robots cannot directly compete with human labor. Harvesting robotic systems do not perform as intended due to two major problems: localization of the object to be harvested because leaves, fruits and other parts of the plant obstruct the view of the robot; and damage to the fruit due to the sensitive nature of agricultural products. In the future, the performance of harvesting robots should be comparable to those of human workers. In order to do so, they should be able to deal with clusters of fruit and occlusions (better software), and harvest the product reliably without damaging it (hardware). Faster and more accurate vision algorithms should be developed, that work together with soft, conflict-free end effectors, as well as manipulators suitable for trees. Already, various papers are trying to address the above-mentioned problems, for example, Cornell University recently published a paper presenting a deep learning method that improves the performance of a robotic arm end-effector in unstructured and occluded environments [127], and at the same time there is research ongoing for novel end-effector designs that fit the needs of specific crops [128]. At this point it is worth mentioning that it is essential for developers to work closely with horticulturists to come up with suitable tree training methods, that facilitate robotic harvesting and at the same time provide high yield and high-quality products. Finally, the picking speed which appears low could be overcome if the robots are able to work for an extensive period of time. Their overall performance can also be solved if human workers harvests the ends of the field and robots the inner parts, to increase their speed. 

### 3.7. Plant Management Robots

In addition to the main above-mentioned field operations, robots that focus on plant architecture have been included. More specifically, they focus on material handling, pruning/thinning, and string twining. Here, the robots that have been developed for these types of field tasks are presented (Table 8).

Material handling is not a task of high importance in the production cycle, however it is crucial in growing operations such as plant nurseries. Therefore, Harvest Automation Inc. developed an electric, fully autonomous material handling robot for unstructured environments (e.g., greenhouses, hoop houses, etc.) [129]. 

As has already been indicated, high-value crops get most of the attention, and one of the most studied crops is grapevines. Subsequently, both pruning robots under review focus on grapevines. The first one was developed by a commercial company titled Vision robotics [130], and therefore no technical information is available, while the second one was developed by the University of Canterbury. In order to effectively prune grapevines, the robot would completely cover the plant to block the sunlight, and then use a six-jointed robot arm mounted with a cutter, and three-color cameras in combination with LED lights [131]. Similarly, a robotic end-effector for the pruning of apple trees resulted in the branch cutting force being in a logarithmic relationship with the branch diameter [132].

String twining is a labor-intense task in crops such as high trellis hop, and there is a high demand for mechanization of this operation. To this end, a string twining robot has been developed, which achieved a success rate up to 97% while was moving with speed of 0.19 m s^−1^ [133].

In conclusion, due to the limited number of publications for each task included in this section, the challenges that have to be dealt with in the future are not clear and solid. However, pruning robots should be able to deal with specific key challenges related to crop architecture, such as height and branches diameter. In this light, suitable sensors should be available on the platform; i.e., force sensors, a vision system, a multi-degrees of freedom end-effector, and, in the background, algorithms that will decide the optimal pruning per plant. Finally, the key challenges that string twining robots face are connected mostly to their performance (faster operational speed and success rate), which is directly connected with the vision system, the end-effector, and the overall platform performance. Overall, there should be more experimental and research studies in the future regarding plant management and architecture robots, in order to optimize their sensing parts and software. In this light, in the current systems, faster execution time cycles could be achieved by adapting higher precision vision systems and faster algorithms.

### 3.8. Multi-Purpose Robotic Systems

Within the crop production cycle, most field operations are executed over short periods of time and only once or twice per year. Additionally, those time spans are usually the same for all growers. As a result, buying and sharing a robot is not a feasible option. One of the widely accepted solutions, to reduce the depreciation time of machinery and make it affordable to growers, is to add value to the robotic system mostly by designing it for more than one field operation. This approach has been adopted by universities, research centers and commercial companies. In order to allow the robot to execute more than one task, three main approaches have been identified: designing a robot that can be equipped with different implements [134], designing a modular robot [135], or equipping the robot with all the tools from the beginning [136]. It is worth mentioning that all robots are autonomous, however, two of them allow the user to manually control them as well if needed [137,138], and two of them are fixed in place with teleoperation systems being used [139,140]. A list of the most significant reviewed multi-purpose robots and the tasks that they can perform is given in Table 9.

Different design approaches lead to robots that are equipped with a variety of perception and measurement sensors. There are robots equipped only with humidity and temperature sensors [136], robots that combine more sensors (humidity, temperature sensors and color cameras) [139] and robots that are just equipped with color cameras [134]. Finally, as mentioned before, some of the robots are modular, meaning the sensors that they carry change according to the modules used [141], while other robots can be fitted with additional sensors according to the growers’ needs [134].

Most of the multi-purpose robots presented above do not include specific performance metrics. A smart robotic machine equipped with a computer vision system for weeding and variable-rate irrigation schemes presented up to 90% weeding rate and 75% wet distribution area [142]. Similarly to weed detection, fungal disease detection, combined with treatment operations, presented significant fungicide savings (85%) when automatic detection was used [143]. Furthermore, more than 97.7% of maize seedlings were accurately detected, in order to prepare maize fields for tillage operations [144]. 

To conclude, multipurpose robots perform more than one field operation of the production cycle, and at this point in time, no solutions have achieved the level of modularity and good performance needed, as being able to perform multiple tasks adds complexity to hardware and software. On the bright side, the same solutions that can be used for the individual task robots can also be applied to the multipurpose ones. Besides, for those problems and solutions that have already been mentioned in previous chapters, multipurpose robots have to perform adequately/accurately for each and every task they are developed for. In addition, the ability to fulfill more than one task increases the cost of the robot, due to complex configurations and the variety of sensors needed. A way to justify higher costs is by extending their functionalities using relatively cheap sensors to measure additional parameters.

## 4. Discussion

**Figure 3:**
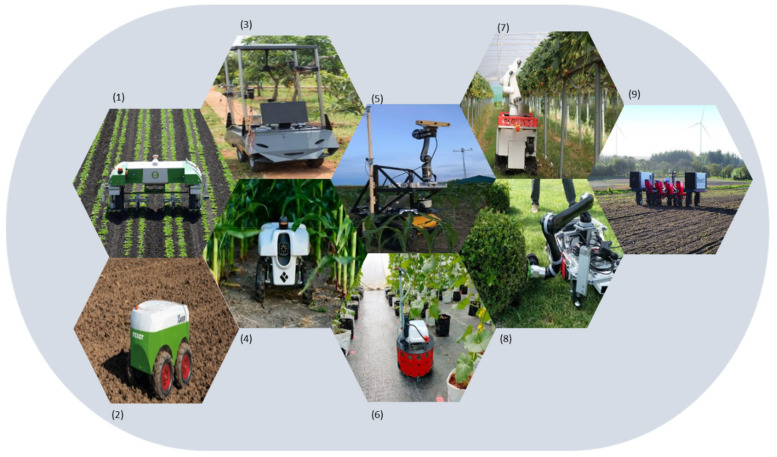
Representation of various types of agricultural robots ((**1**) weeding [23], (**2**) seeding [47], (**3**) disease and insect detection [55], (**4**) plant monitoring [69], (**5**) phenotyping [78], (**6**) spraying [86], (**7**) harvesting [100], (**8**) plant management [152], (**9**) multi-purpose [137]).

The reviewed literature of robotic systems in agricultural field operations was classified according to major field operations; weeding, seeding, disease and insect detection, crop scouting, spraying, harvesting, plant management, and multi-purpose robots. An indicative representation of each category robot is depicted in Figure 3.

The number of references included in this review was 153 in total. They have been allocated, according to the referenced sources, to four main categories, namely research journal sources, conference proceedings, commercial and other sources (such as EU-projects, book chapters, etc). The research journal studies were retrieved from various journals, such as “Computers and Electronics in Agriculture”, “Biosystems Engineering”, “Precision Agriculture”, “Journal of Robotics”, “International Journal of Agricultural and Biological Engineering”, etc. In addition to this, part of these references have been published in conference proceedings, while others are related to commercial robots and sources such as international research projects. 

**Figure 4:**
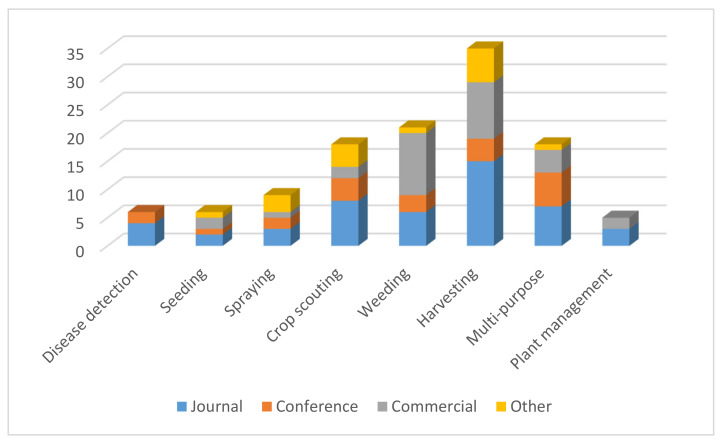
Number of reviewed robots per field operation.

Agricultural robots have been recognized as one of the most promising solutions for solving the challenges agriculture is currently facing. Agricultural robots have been developed for every operation in the crop production cycle, starting with the establishment of a crop by seeding, and ending with the harvesting of the final agricultural product. However, the tasks that have attracted most of the attention are the most labor-intensive ones, namely harvesting and weeding. As most of the agricultural tasks are, in principal, seasonal, within a specific period of time, the only way to allow for a quick return of investment (ROI)—which is the most convincing argument for a grower to invest—is by adding value to the robot by allowing it to perform more than one task, which explains why multi-purpose robots have been recently brought into the spotlight. The extent to which the ROI and labor can affect the development of robots is apparent, especially when comparing the number of commercial robots for each task and the number of robots developed by academia. Companies focus on the two operations mentioned above, weeding and harvesting, while academia focuses mostly on crop scouting, weeding, harvesting and multipurpose. Harvesting and multipurpose tasks usually require a complicated solution that attracts a lot of attention, and therefore provides exposure of the university or research center to a wider part of the population. All of the above can be clearly seen in Figure 4.

In all these operations there are specific issues that have to be tackled. Regarding the communication issue, which applies in all agricultural robots, the focus is on the way data is transmitted between the various components or the way this information reaches the user. The most popular technologies that address this challenge are WiFi [22,37,56,70,78], Ethernet [63,73,75,83,107,123], CAN-bus [13,22,27,38,75,108,153], and RS-232 [59,63,71,85,134]. For seeding robots, the optimal working speed according to crop and field features, and the image detection accuracy, are the key factors that should be further improved. A variety of sensors have been used to increase precision and speed in the seeding operation, such as RGB cameras, force, displacement and pressure, infrared sensors, and wheel encoders. However, there is still research to be carried out for better fusion of sensors, and potentially novel sensors and techniques should be tested, e.g., spectroscopy to estimate soil texture for the optimum seed depth and space placement. Weeding robots (chemical or mechanical) have been widely developed in the last years, and they offer the most commercially available systems, as weeding is tedious and labor-intensive work, especially for weeding within the crop row. However, there are optimization issues that should be faced, i.e., robots provide a small working width and slow working speed, restricting the deployment in large-scale farms and higher accuracy weed detection for selective spot spraying. So far, weeding robots have been using, in the vast majority, RGB cameras in combination with a sensor to identify the distance from the object, such as optical and acoustic distance sensors, laser range finders or stereo vision systems. Weeding robots should also be assessed and customized to be able to operate in crops with similar operational requirements. 

For disease and insect detection robots, three core issues have been identified: the lack of image databases; the slow image processing (for large volumes of images); and the non-uniformity of field lighting conditions. However, these issues are gradually addressed by the use of available open-access image databases, by applying new methods on data synthesis processing, and by the use of innovative lighting systems. Similar illumination-related issues have been presented in monitoring and phenotyping robots, and they are faced by artificial lighting systems. Another challenge for the monitoring and phenotyping robots is the separation of the background. This issue has been addressed by using wider rows in the field, or by implementing other technological approaches, such as imaging of the same plant from multiple angles, and the development of better and faster algorithms. It is obvious that the amount of generated data should be efficiently handled by suitable dataset processing, automated workflows and pipelines. Spraying robots have presented similar issues to disease detection robots. Additional problems are the low spraying tank capacity and the autonomy of the robot. Even though the working speed may be considerably low, as not all plants need to be sprayed, the total spraying time of selective sprayers can be lower than conventional non-selective ones. In addition, selective sprayers can reduce drift and runoff using micro sprayers. In this light, suggested solutions include more spray nozzles, multiple operating platforms and, of course, increased speed of detection algorithms. All the above-mentioned task-specific robots use optical sensors to be able to perform their tasks, mainly RGB cameras, multi-/hyper-spectral cameras, and LiDAR sensors, therefore as optics technology advances, so will the performance of the robots.

Harvesting robots have gained wide interest globally, even though they should be further optimized. Two major problems have been underlined: the localization of the object to be harvested because leaves, fruits and other parts of the plant obstruct the view of the robot; and the potential damage to the fruit due to the sensitive nature of agricultural products. In order to develop harvesting robots with performance comparable to human workers, they should be equipped with better software and hardware; better software to deal with clusters of fruit and occlusions, and better hardware to operate harvesting reliably, without causing damages to the product and the plant. In order to harvest the ripe fruit, a vision system is needed to evaluate the maturity, and locate and grasp the fruit, and as a result, all harvesting robots include a camera (RGB, Time-of-Flight, 3D or Stereo camera) and an additional sensor to measure distance if the camera cannot provide that, such as a LiDAR sensor. Moreover, faster and more accurate vision algorithms should be developed, that work together with soft, conflict-free end effectors, as well as manipulators suitable for trees.

For the plant management robots, due to the limited number of references for each task, the challenges that have to be dealt with in the future cannot be outlined and summarized. However, given the challenges that other task-specific robots face, it could be stated that both software (e.g., better machine vision algorithms) and hardware (e.g., lightweight platforms and tools) development are needed in order to improve current robots’ performance. Moreover, further scientific research and experimental trials should be held, targeting the development of new robotic solutions in other agricultural tasks where no robotic systems exist.

Multipurpose robots perform more than one field operation, and up to now, there have been no solutions for achieving the required level of modularity and good performance. Besides those problems and solutions that have already been mentioned in the single-task robots, multipurpose robots have to perform adequately/accurately for each task they have been developed for. More tasks means more complexity hardware-wise and software-wise, and this should be a tech improvement for future robots. Because of the need to perform more than one task, the sensors used by these multipurpose robots should have a universal value; this leads to the wide use of optical sensors, such as IR and RGB cameras, in combination with more task-specific ones, like humidity, temperature and luminosity sensors. Generally, the ability to fulfill more than one task increases the cost of the robot, due to complexity of configurations and the variety of components needed. A way to justify the higher costs is by extending their functionalities and by making them compatible with various groups of crops.

**Figure 5:**
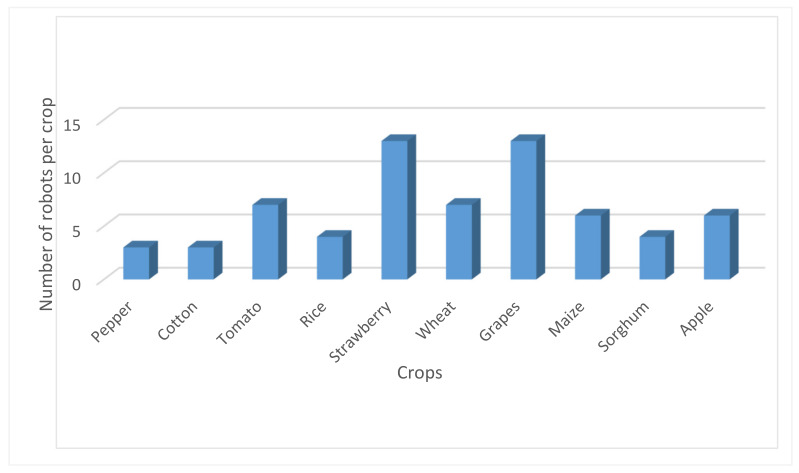
The main crops in correlation with the number of robotic systems.

Agricultural robots are associated with high investment costs from the farmer’s point of view. Consequently, there is a need for new business models in agriculture, such as robotic services contractors. As has been mentioned above, a way to return the investment fast is by investing in multi-purpose agricultural robots. In addition to this, another way to enable a quick ROI is to target high-value crops that pay back the amount invested. Furthermore, the crops that the robot will operate must require extensive crop care in order to justify replacing humans. As a result, most of the robots have been developed under this scope, with strawberries and grapes being the most popular amongst the agro-robotics community (Figure 5).

**Figure 6:**
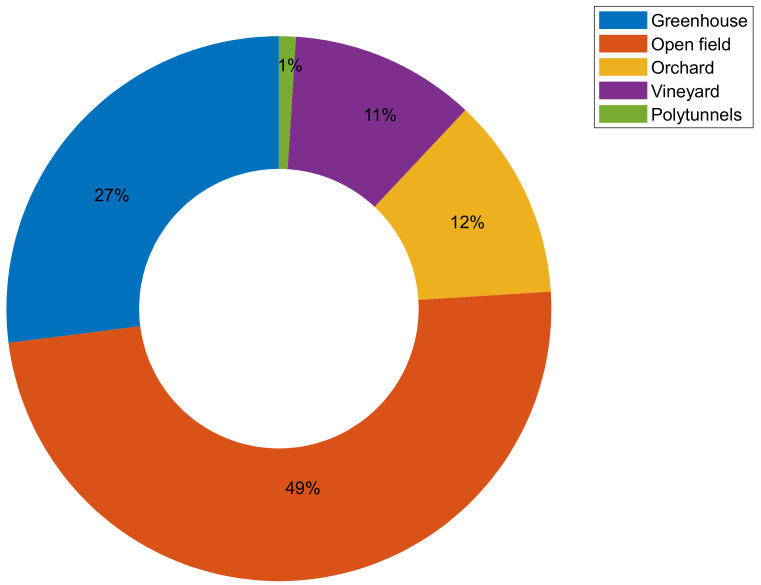
Allocation (%) of robotic systems in various agricultural environments.

Agriculture is one of the more unstructured environments, with constant changes, and most systems are not designed in an adaptive way; therefore it is hard to robotize/automate tasks. Moreover, only recently have learning systems been able to adapt to new situations, including agriculture. Plant growth cannot be predicted accurately, obstacles may appear suddenly (animals, rocks, etc.), and occlusions are a common problem due to the growth of the plant and movement of branches. Finally, lighting conditions are constantly changing, due to clouds and the day/night cycle. As a result, one would assume that robots operating in a semi-structured environment, such as greenhouses or orchards and vineyards, would be developed as front-runners. Nevertheless, that is not the case, as almost half of the developed robots focus on open field crops (Figure 6). This could be attributed to the fact that the majority of the crops worldwide are cultivated as open field crops, or to the fact that weeding, which is one of the most popular robot categories, is mainly an issue in open fields and not greenhouses.

## 5. Conclusions

Overall, throughout all the reviewed robots, the most-studied robot categories were the harvesting and weeding robots, while the least-studied were disease detection and seeding robots. From the studied robots, specific and universal conclusions can be pointed out in order to address the current key issues and potential future challenges. A general overview of an agricultural robot includes part or all of the following components: a navigation system; a vision system; a control system; communication components; the robotic arm with its components; and, of course, a computer, a safety system, a remote assistance/tele-robotics system (to assist the robot when in failure mode), an edge/cloud-AI adaptive learning system and a farmer-friendly brilliant simple interface system. The majority of the robots make use of RTK-GPS for navigation, and LiDAR in order to reconstruct the environment digitally. Moreover, RGB, stereo and multi- and hyper-spectral cameras have been widely used, to allow the localization of important environmental objects and features, so that the robot can perform its task consistently and with high accuracy. Better vision systems are needed for all operations in parallel with faster image processing methods/algorithms. In particular, image processing is directly connected with processing methods, communication means, vision systems and the volume of the images. A key challenge related to the robotic vision systems is to provide uniform lighting conditions by artificial illumination means. Furthermore, new deep learning methods could be developed to improve the performance of the robotic arm end-effectors, and, more specifically, improve the eye-hand coordination of the robot by using learning methods (such as Grasp Quality Convolutional Neural Network). As for the computer hardware, it should be able to deal with the huge amount of data quickly, especially where high-resolution images are needed.

## Figures and Tables

**Table 1:** Weeding agrirobotic systems’ characteristics for various crops.

Crop	Perception Sensors	Weed Detection	Weed Control	Results	Cited Work
Maize	Cameras, optical and acoustic distance sensors	Yes	Chemical	No performance metrics provided	[38]
Carrot	RGB infrared camera	Partly	Chemical	100% effectiveness with the DoD system	[22]
Potato, corn	Webcam, solid-state gyroscope	Partly	Chemical	98% and 89% detection accuracy	[36]
Sugar beet	Color camera	Yes	Mechanical	Row detection precision < 25 mm. > 90% in-row weed removal	[24]
N/A	Stereo vision system, laser	No	Mechanical	Precision < 3 cm.	[27,29]
Rice	Laser range finder, IMU	No	Mechanical	Precision < 62 mm	[25]
Beetroot	Color camera, artificial vision, compass	Yes	Chemical	> 85% detection & destroy, precision < 2 cm	[37]
Grapes	IMU, hall sensors, electromechanical sensor, a sonar sensor	No	Mechanical	Average performance: 65% (feeler) & 82% (sonar)	[30]
N/A	Accelerometer, gyroscope, flex sensor	No	Mechanical	No performance metrics provided	[31]
Tomato	Color camera, SensorWatch	Partly	Chemical	24.2% were incorrectly identified and sprayed and 52.4% of the weeds were not sprayed.	[42]

**Table 2:** Seeding agrirobotic systems’ characteristics for wheat and rice.

Crop	Perception	Results	Cited Work
Wheat	Force sensor, displacement sensor, angle sensor	The path tracking errors are +/− 5 cm and the angle errors are about zero.	[49]
Wheat	Signal sensor, angle, pressure & infrared sensors	Qualified rate: 93.3%	[44]
Rice	Compass, wheel encoder	92% accuracy & 5 cm error in the dropping position.	[45]

**Table 3:** Disease and insect detection agrirobotic systems features for various crops.

Crop	Perception	Detected Disease	Highest Accuracy	Cited Work
Bell pepper	RGB camera, multispectral camera, laser sensor	Powdery mildew & tomato spotted wilt virus	95% & 90%	[51,52]
Cotton, groundnut	RGB camera	Cotton (Bacterial blight, magnesium deficiency), groundnut (leaf spot & anthracnose)	~90%, 83–96%	[56]
Olive tree	Two DSLR cameras (one in BNDVI mode), a multispectral camera, a hyperspectral system in visible and NIR range, a thermal camera, LiDAR, an IMU sensor^(^*^)^	*Xylella fastidiosa* bacterium	N/A	[53]
Tomato, rice	RGB camera	*Pyralidae* insect	94.3%	[55]
Strawberry	RGB camera	Powdery mildew	72%-95%	[54]

^(^*^)^DSLR: Digital Single-lens Reflex. BNDVI: Blue Normalised Difference Vegetation Index. NIR: near-infrared. LiDAR: Light detection and ranging.

**Table 4:** Plant vigor monitoring agrirobotic systems for various crops.

Crop	Perception	Results	Cited Work
No specific crop	High-resolution stereo-cameras, 3D LiDAR	Soil sampling. No performance metrics provided.	[70]
No specific crop	CO_2_ gas sensor, anemoscope, IR distance measuring sensor	Gas source tracking. CO_2_ concentration levels up to 2500 ppm were recorded while the robot was moving at a speed of 2 m/min.	[71]
Orchards	LiDAR, luxmeter	Canopy volume estimation. The system is independent of the light conditions, it is highly reliable and data processing is very fast.	[62]
Grapes	RGB & IR camera, laser ranger finder, IMU, pressure sensor, etc.	Crop monitoring tasks. No performance metrics provided.	[68]
Orchards and vineyards	LiDAR, OptRX sensor	Monitor health status and canopy thickness. Terrestrial Laser Scanning (TLS): 2 mm distance accuracy	[63]
Canola	Ultrasonic sensors, NDVI sensors, IR thermometers, RGB camera	Gather phenotypic data. Maximum measurement error: 2.5%	[59]

**Table 5:** Phenotyping agrirobotic systems for various crops.

Crop	Perception	Autonomy Level	Results	Cited Work
Maize & wheat	Cameras, spectral imaging systems, laser sensors, 3D time-of-flight cameras	Autonomous	No performance metrics provided	[75]
Cotton	Stereo RGB & IR thermal camera, temperature, humidity, light intensity sensors, pyranometer, quantum, LiDAR	Semi-autonomous	RMS error: Plant height: < 0.5 cm (Vinobot) RGB to IR calibration: 2.5 px (Vinoculer) Temp: < 1 °C (Vinoculer)	[78]
Sorghum	Stereo camera, RGB camera with fish eye lenses, penetrometer	Autonomous	Stalk detection: 96%	[73]
Rice, maize & wheat	RGB camera, chlorophyll fluorescence camera, NDVI camera, thermal infrared camera, hyperspectral camera, 3D laser scanner	Fixed site fully automated	Plant height RMS error: 1.88 cm	[76]
Sugar Beet	Mobile robot: Webcam camera, gigaethernet camera Bettybot: Color camera, hyperspectral camera	Autonomous	No performance metrics provided	[74]
Sorghum	Stereo imaging system consisting of color cameras	Autonomous based on commercial tractor	The image-derived measurements were highly repeatable & showed high correlations with manual measurements.	[77]
Energy Sorghum	Stereo camera, time of flight depth sensor, IR camera	Semi-autonomous	Average absolute error for stem width and plant height: 13% and 15%.	[79]

**Table 6:** Spraying agrirobotic systems’ features for various crops.

Crop	Perception	Real-time Detection	Results	Cited Work
Cantaloupe	Robot controller	No	NSGA-II execution time was better 1.5–7% than NSGA-III for the same test cases. ^(^*^)^	[86]
N/A	Web camera	Yes	27% off-target shots, 99.8% of the targets were sprayed by at least one shot.	[90]
Cucumber	Bump sensors, infra-red sensors, induction sensors	No	Run success:90% & 95%, topside leaf coverage:95% & 90%, underside leaf coverage:95 & 80%, over-spray:20% & 10% for Tests 1 & 2	[83]
N/A	Ultrasonic sensors	No	Maximum error varied from 1.2–4.5 cm for self-contained mode on concrete, while for trailer varied from 2.2–4.9 cm.	[87]
Grapevine	Ultrasonic sensor, color TV camera	No	No performance metrics.	[91]
N/A	Middle-range sonar, short-range sonar, radar, compass, radar	No	Longitudinal, lateral and orientation errors are close to zero, by including slip varied 10–30%.	[85]
Grapevine	RGB camera, R-G-NIR multispectral camera	Yes	The sensitivity of the robotic selective spraying was 85%, the selectivity was 92% (8% of the total healthy area was sprayed unnecessarily).	[88]
Vegetable crops	Hyperspectral camera, stereo vision, thermal IR camera, monocular color camera	Yes	The greedy sort and raster methods are substantially faster than back-to-front scheduling, taking only 68% to 77% of the time.	[89]

^(^*^)^NSGA: Non-dominated Sorting Genetic Algorithm.

**Table 7:** Harvesting agrirobotic systems’ features for various crops.

Crop	Perception	Fastest Picking Speed	Highest Picking Rate	Cited Work
alfalfa, sudan	Color camera, gyroscope	2 ha/h (alfalfa)	N/A	[123]
apple tree	Color camera, time of flight based three dimensional camera	7.5 sec/fruit	84%	[103]
apple tree	Color CCD (Charge Coupled Device) camera, laser range sensor	7.1 sec/fruit	89%	[105]
apple tree	High-frequency light, camera	9 sec/fruit	80%	[104]
cherry	3D vision sensor with red, IR laser diodes, pressure sensor	14 sec/fruit	N/A	[113]
mushroom	Laser sensor, vision sensor	6.7 sec/mushroom	69%	[124]
asparagus	3D vision sensor with two sets of slit laser projectors & a TV camera	13.7 sec/asparagus	N/A	[118]
strawberry	Sonar camera sensor, binocular camera	31.3 sec/fruit	86%	[94]
strawberry	Color CCD cameras, reflection-type photoelectric sensor	8.6 sec/fruit	54.9%	[92]
strawberry	LED light source, three-color CCD cameras, photoelectric sensor, suction device	11.5 sec/fruit	41.3% with a suction device 34.9% without it	[98]
strawberry	Color CCD camera, visual sensor	10 sec/fruit	N/A	[97]
strawberry	Three VGA (Video Graphics Array) class CCD color cameras (stereo vision system and center camera)	N/A	46%	[99]
strawberry	RGB-D camera, 3 IR sensors	10.6 sec/fruit	53.6%	[95]
tomato	Stereo camera, playstation camera	23 sec/tomato	60%	[107]
tomato	Binocular stereo vision system, laser sensor	15 sec/tomato	86%	[108]
cherry tomato	Camera, laser sensor	8 sec/tomato bunch	83%	[109]
cucumber	Two synchronized CCD cameras	45 sec/cucumber	80%	[115]
various fruits	Pressure sensor, 2 convergent IR sensors, telemeter, cameras	2 sec/fruit (only grasp & detach)	N/A	[114]
melon	Two black and white CCD cameras, proximity sensor, far and near vision sensors	15 sec/fruit	85.67%	[121]
eggplant	Single CCD camera, photoelectric sensor	64.1 sec/eggplant	62.5%	[116]
watermelon	Two CCD cameras, vacuum sensor	N/A	66.7%	[122]

**Table 8:** Plant management agrirobotic systems for various crops.

Crop	Perception	Operation	Cited Work
Apple tree	Force sensor	pruning	[132]
Hop	Camera, wire detecting bar	string twining	[133]
Grape	Three cameras	pruning	[131]

**Table 9:** Multi-purpose agrirobotic systems for various crops.

Working Environment	Perception	Operation(s)	Cited Work
Arable crops	N/A	Ploughing, seeding	[145]
Arable crops	Sonar sensor, temperature	Monitoring, spraying, fertilization, disease detection	[138]
Arable crops	Τhree cameras with IR filter, humidity & ultrasonic sensors	Ploughing, seeding, harvesting, spraying	[146]
Arable crops, polytunnels, greenhouse	2D LiDAR, ultrasonic sensors, an RGB camera & a monochromatic IR camera	Harrowing, soil sampling, phenotyping, additional tasks by combining modules	[135]
Arable crops	Soil sensors	Ploughing, irrigation, seeding	[147]
Greenhouse	Two color cameras, machine vision sensor	Spraying, weeding, additional tasks by adding or removing components	[134]
Arable crops	Humidity and temperature air sensor, IR sensors	Ploughing, seeding, irrigation, spraying, monitoring	[136]
Urban crops	Color camera, temperature, humidity and luminosity sensors	Sowing, irrigation, fumigation, pruning	[139]
Arable crops	Voltage sensors	Seeding, spraying, ploughing, mowing	[148]
Arable crops	2D LiDAR, ultrasonic sensor, IR camera	Seeding, weeding, ploughing	[141]
Arable crops	N/A	Sowing, sprinkling, weeding, harvesting	[149]
Arable crops	CloverCam, RoboWeedCamRT	Seeding, weeding	[137]
Vineyards	N/A	Pruning, weeding, mowing	[150]
Arable crops	N/A	Precision seeding, ridging discs & mechanical row crop cleaning	[151]
